# Stellenwert der molekularen Testung von Schilddrüsenpunktaten

**DOI:** 10.1007/s00292-021-01049-x

**Published:** 2022-01-10

**Authors:** O. Chijioke

**Affiliations:** Institut für Pathologie, Universitätsspital Basel, Schönbeinstraße 40, 4031 Basel, Schweiz

**Keywords:** Feinnadelpunktion, Genetische Alteration, Prädiktive Marker, Schilddrüsenkarzinom, Schilddrüsenknoten, Fine-needle biopsy, Genetic alteration, Predictive markers, Thyroid carcinoma, Thyroid nodule

## Abstract

Zur Abklärung von Schilddrüsenknoten spielt die zytologische Untersuchung von Feinnadelpunktaten der Schilddrüse eine zentrale Rolle. Dabei sollten etablierte Klassifikationsschemata zur Anwendung kommen. Bei unklaren zytologischen Befunden können molekulare Zusatzuntersuchungen eingesetzt werden. Der Stratifizierung unklarer Schilddrüsenknoten in maligne und benigne Läsionen allein anhand molekularer Testverfahren sind jedoch abgesehen von kostspieligen kommerziellen Tests US-amerikanischer Anbieter bislang klare Grenzen gesetzt. Hilfreich und relativ einfach durchzuführen sind molekulare Tests einzelner genetischer Alterationen, die die Malignität bei papillären, gering differenzierten und anaplastischen Schilddrüsenkarzinomen bestätigen können. Negative Testresultate schließen dabei jedoch eine maligne Neoplasie keineswegs aus. Prädiktive Marker für einzelne Entitäten (*BRAF* V600E, *RET*-Mutationen und *RET*-Fusionen) sollten bei allen fortgeschrittenen Schilddrüsenkarzinomen getestet werden.

## Abstract (en)

In the evaluation of thyroid nodules, cytopathology of thyroid fine-needle aspiration specimens plays a central role. Established classification schemes should be used. In the case of indeterminate cytology, additional molecular tests may be used. However, the stratification of indeterminate thyroid nodules into malignant and benign lesions based on molecular tests alone, apart from costly commercial assays from US vendors, has so far been clearly limited. Molecular testing of single genetic alterations that can confirm malignancy in papillary, poorly differentiated, and anaplastic thyroid carcinomas is helpful and relatively easy to perform. However, negative test results by no means exclude malignant neoplasia. Predictive markers for single entities (*BRAF* V600E, *RET* mutations and *RET* fusions) should be tested in all advanced thyroid carcinomas.

Schilddrüsenknoten sind weit verbreitet und weisen eine mit dem Alter zunehmende Prävalenz auf [11, 19]. Der überwiegende Anteil der Schilddrüsenknoten ist benigner Natur und entspricht meist Adenomen oder Hyperplasien. Nur ca. 5 % der Schilddrüsenknoten sind maligne, wobei davon aufgrund steigender relativer Inzidenz bis zu 90 % papilläre Schilddrüsenkarzinome ausmachen, gefolgt von follikulären, medullären, gering differenzierten und anaplastischen Schilddrüsenkarzinomen [7, 15].

Ziel einer Schilddrüsenpunktion ist die Abklärung von Schilddrüsenknoten und das Abschätzen des Malignitätsrisikos. Die zytologische Diagnostik von Schilddrüsenpunktaten nimmt somit neben der Bildgebung [20, 25] einen zentralen Stellenwert für die klinische Wegleitung ein. Das Bethesda-System zur Befundung der Zytopathologie der Schilddrüse (TBSRTC), das erstmals 2009 publiziert [5] und 2017 überarbeitet [6] wurde, dient als ein weitverbreitetes Bezugssystem der Vereinheitlichung und Kategorisierung (Kategorien I bis VI) der zytologischen Diagnostik von Feinnadelpunktaten der Schilddrüse (Tab. 1).

**Table 1:** 

*Diagnostische Kategorie*	*Beschreibung*
I	Nicht diagnostisch oder unzulänglich
II	Benigne
III	Atypie unklarer Signifikanz oder follikuläre Läsion unklarer Signifikanz
IV	Follikuläre Neoplasie oder verdächtig auf follikuläre Neoplasie
V	Verdächtig auf Malignität
VI	Maligne

Da die Entität der nichtinvasiven follikulären Neoplasie mit dem papillären Schilddrüsenkarzinom äquivalenten Kernmerkmalen (NIFTP) [16, 17] nicht mehr den Schilddrüsenkarzinomen zugeordnet wird, hat sich das Malignitätsrisiko für die Kategorien III bis VI verringert (Tab. 2; [6, 21]). Allerdings verweisen große Registerstudien (noch ohne Beachtung der NIFTP) darauf, dass das Malignitätsrisiko je nach Einschluss der klinisch fraglich relevanten Mikrokarzinome höher liegen kann als ursprünglich im Bethesda-System beschrieben (Tab. 2; [12]).

**Table 2:** 

Diagnostische Kategorie [6]	Häufigkeit (%) [12]	ROM (%), TBSRTC [6]		ROM (%), ohne MK [12]	ROM (%), mit MK [12]	PPV (%) [12]	NPV (%) [12]
		NIFTP = benigne	NIFTP = maligne				
I	6,7	5–10	5–10	13,6	19,3	–	–
II	32,4	0–3	0–3	7,8	12,7	40,7	80,7
III	14,5	6–18	10–30	24,5	31,9	55,6	86,2
IV	21,6	10–40	25–40	26,7	31,4	63	81,3
V	7,7	45–60	50–75	73,7	77,8	90,4	77,7
VI	17,2	94–96	97–99	95,4	96	96	72,5

*MK* Mikrokarzinom, *NIFTP* nichtinvasive follikuläre Neoplasie mit dem papillären Schilddrüsenkarzinom äquivalenten Kernmerkmalen, *NPV* negativer prädiktiver Vorhersagewert, *PPV* positiver prädiktiver Vorhersagewert, *ROM* „risk of malignancy“ (Malignitätsrisiko)

Für die Entscheidungsfindung in der weiteren Behandlungsplanung bereiten insbesondere die Kategorien III (Atypien unklarer Signifikanz oder follikuläre Läsion unklarer Signifikanz) und IV (follikuläre Neoplasie oder Verdacht auf follikuläre Neoplasie) Schwierigkeiten, insofern sich daraus keine klaren Handlungsanweisungen für die Klinik eröffnen. Um rein diagnostische chirurgische Eingriffe zu vermeiden, wird sich Abhilfe von molekularen Untersuchungen erhofft, die eine maligne Erkrankung mit möglichst hoher Treffsicherheit ausschließen sollen (Rule-out-Test) und somit ein nichtchirurgisches, beobachtendes Vorgehen rechtfertigen. Andererseits sollen molekulare Testverfahren dazu dienen, eine maligne Erkrankung mit großer Wahrscheinlichkeit bestätigen zu können (Rule-in-Test). Um eine Einschätzung des Potenzials solcher Tests zu ermöglichen, wird im Folgenden das genetische Profil verschiedener Neoplasien der Schilddrüse beschrieben.

## Genetische Alterationen in neoplastischen Läsionen der Schilddrüse

Die häufigste maligne Neoplasie der Schilddrüse mit bis zu 90 % aller Schilddrüsenkarzinome ist das papilläre Schilddrüsenkarzinom [7, 15]. Die wichtigsten genetischen Alterationen der papillären Schilddrüsenkarzinome sind *BRAF* V600E-Mutationen (ca. 60 %) sowie Fusionen von *RET* (ca. 7 %), *NTRK1, NTRK3* oder *ALK* (jeweils ca. 1 %) neben Mutationen in *RAS*-Genen (ca. 13 %; in absteigender Häufigkeit *NRAS, HRAS* und *KRAS*) und *TERT*-Promoter-Mutationen (10 %) [2]. Bei der follikulären Variante des papillären Schilddrüsenkarzinoms steigt der relative Anteil an *RAS*-Mutationen (ca. 30 %) gegenüber *BRAF* V600E-Mutationen [30], wohingegen die NIFTP häufig *RAS*-Mutationen sowie *EIF1AX-, DICER1-* und *PTEN-*, jedoch keine *BRAF* V600E-Mutationen aufweisen [3].

Follikuläre Schilddrüsenkarzinome sind anhand des genetischen Profils nicht von follikulären Adenomen abgrenzbar, da sich in beiden neoplastischen Entitäten sowohl Mutationen in *RAS-, DICER1-, EZH1-* und *EIF1AX*-Genen als auch *PAX8*-*PPARG*-Fusionen nachweisen lassen [29]. Die seltenen und den follikulären Neoplasien zugeordneten Hürthle-Zell-Karzinome weisen Mutationen in mitochondrialen Genen und charakteristische chromosomale Kopienzahlveränderungen auf sowie weitere, breit gestreute Mutationen, die jedoch auch in Hürthle-Zell-Adenomen und sogar nichtneoplastischen Läsionen mit onkozytärer Differenzierung beschrieben sind [8, 9]. Jeweils ca. 25 % der gering differenzierten Schilddrüsenkarzinome sind *BRAF* V600E- oder *RAS-*mutiert und ca. 40 % zeigen *TERT*-Promoter-Mutationen [27], wohingegen anaplastische Schilddrüsenkarzinome auf molekularer Ebene gekennzeichnet sind durch einen hohen Anteil an *BRAF* V600E- (ca. 45 %), *TERT*-Promoter- (ca. 70 %) und *TP53*-Mutationen (ca. 70 %) [13]. In nahezu sämtlichen hereditären medullären Schilddrüsenkarzinomen finden sich *RET*-Mutationen [7]. Hingegen weisen die weitaus häufigeren sporadischen medullären Schilddrüsenkarzinome nur in ca. 60 % *RET-*Mutationen auf und ca. 30 % zeigen Mutationen in *RAS*-Genen [4].

## Stellenwert molekularer Untersuchungen von Schilddrüsenpunktaten

Bei der Abklärung von unklaren zytologischen Befunden (Kategorien III und IV des Bethesda-Systems) verweisen verschiedene Studien auf den Nutzen zusätzlicher molekularer Untersuchungen anhand kommerzieller Tests im Rule-out-Verfahren, um Malignität auszuschließen und unnötige chirurgische Eingriffe zu vermeiden [14]. Die meisten der kommerziellen Testsysteme sind jedoch hochkomplex, kostspielig [18] und werden in europäischen Ländern kaum verwendet. Testverfahren, die einen Ausschluss einer maligen Neoplasie der Schilddrüse durch molekulare Analysen ermöglichen, sind in Europa bislang nicht etabliert. Bestrebungen auf europäischer Ebene mit Möglichkeit für eine dezentrale Testung sind jedoch in Gange [1, 22].

Genetische Alterationen, die mit sehr hoher Treffsicherheit eine maligne Neoplasie der Schilddrüse vorhersagen und somit als Rule-in-Tests dienen können, sind insbesondere die *BRAF* V600E-Mutation [10], die in papillären, gering differenzierten und anaplastischen Schilddrüsenkarzinomen auftreten, neben Fusionen von *RET, NTRK1/3* oder *ALK*. Diese Alterationen finden sich jedoch in zytologisch meist gut fassbaren Entitäten. Prognostisch ungünstig sind die gehäuft in aggressiven Schilddrüsenkarzinomen beschriebenen *TERT*-Promoter- und *TP53*-Mutationen, deren Nachweis somit für die Planung des Ausmaßes eines chirurgischen Eingriffs von Nutzen sein könnte.

Prädiktive Marker für Alterationen mit signifikanter Prävalenz sind sowohl die *BRAF* V600E-Mutation bei anaplastischen Schilddrüsenkarzinomen [24] als auch *RET*-Alterationen (*RET*-Mutationen bei medullären Schilddrüsenkarzinomen und *RET*-Fusionen bei papillären Schilddrüsenkarzinomen) [23, 26]. Entsprechende molekulare Untersuchungen sollten bei fortgeschrittenem Tumorleiden durchgeführt werden, was auch an zytologischem Material möglich ist, um den Zugang zu einer zielgerichteten Therapie zu ermöglichen.

**Figure 1:**
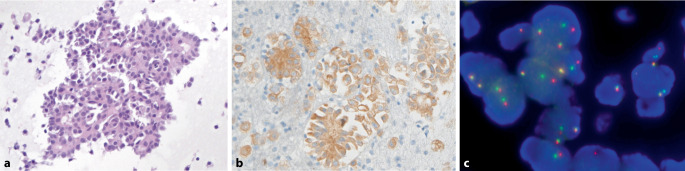

Als molekulare Untersuchungsmethoden am zytologischen Material (Ausstriche oder Zellblöcke) bieten sich somit die Sequenzierung von *BRAF,* beispielsweise via Droplet-digital-PCR, FISH-Untersuchungen von *RET* sowie NGS-Paneluntersuchungen an. Letztere sollten neben Hotspots von *BRAF-* und *RAS*-Genen auch die *TERT*-Promoter-Region und *TP53* abdecken. Ein solches kleineres DNA-basiertes NGS-Panel haben wir in unserer Institution inzwischen mehrfach eingesetzt. Auf Proteinebene können immunhistochemische Färbungen von pan-TRK und ALK als Surrogatmarker für Fusionen genutzt werden. Neben einer immunhistochemischen Färbung auf die *BRAF* V600E-Mutation kann ebenfalls RET als Biomarker in der Immunhistochemie eingesetzt werden mit einer für Schilddrüsenkarzinome kürzlich beschriebenen Sensitivität und Spezifität von jeweils 80 % [28]. Die RET-Immunhistochemie setzen wir inzwischen als Screeningverfahren ein. Bei einem hierfür illustrativen Fall eines metastasierten papillären Schilddrüsenkarzinoms konnten wir damit über eine anschließende FISH-Untersuchung letztlich ein *RET*-Rearrangement nachweisen (Abb. 1).

## Fazit für die Praxis

- Die zytologische Untersuchung von Feinnadelpunktaten der Schilddrüse besitzt einen zentralen Stellenwert für das klinische Management.
- Die zytologische Befundung sollte anhand von etablierten Klassifikationsschemata erfolgen.
- Bei unklaren zytologischen Befunden (Bethesda III und IV) sind molekulare Testverfahren, die einen sicheren Ausschluss von Malignität erlauben, in Europa bislang nicht etabliert.
- Bei follikulären Läsionen sind molekulare Testverfahren nicht geeignet zur Unterscheidung von follikulären Schilddrüsenkarzinomen und follikulären Adenomen.
- Einige wenige genetische Alterationen (*BRAF* V600E, Fusionen von *RET, NTRK1/3, ALK*) sind diagnostisch für Malignität.
- Prädiktive Marker sind bei papillären Schilddrüsenkarzinomen *RET*-Fusionen, bei medullären Schilddrüsenkarzinomen *RET*-Mutationen sowie beim anaplastischen Schilddrüsenkarzinom die *BRAF *V600E-Mutation. Diese sollten bei fortgeschrittenen Tumorleiden getestet werden
